# Choroidal Vascular Density Quantification in High Myopia with or without Choroidal Neovascularization Using Optical Coherence Tomography Angiography

**DOI:** 10.1155/2023/1504834

**Published:** 2023-01-20

**Authors:** Xuehui Lu, Guihua Zhang, Lingping Cen, Yali Du, Lifang Liu, Chuang Jin, Haoyu Chen

**Affiliations:** Joint Shantou International Eye Center, Shantou University & the Chinese University of Hong Kong, Shantou 515041, Guangdong, China

## Abstract

**Purpose:**

The aim of this study was to analyze choroidal vascular density alteration in high myopia with or without choroidal neovascularization by using optical coherence tomography angiography (OCTA).

**Methods:**

This was a cross-sectional, observational study that included 60 high-myopia eyes. All the participants had comprehensive ophthalmic assessments with visual acuity, intraocular pressure, slit lamp-assisted biomicroscopy, color fundus photography, axial length, optometry, and OCTA. Age, sex, and comorbidities were collected from their medical charts. Univariate and multiple analyses were made to compare the age, spherical equivalent, choroidal vascular density, gender, and choroidal thickness between normal and patients with choroidal neovascularization.

**Results:**

60 eyes with high myopia were included in our study, including 30 eyes with choroidal neovascularization and 30 eyes without choroidal neovascularization or other fundus pathology. The mean age of high myopic patients was older in the choroidal neovascularization group than in the normal group (48.43 ± 19.06 years vs. 28.83 ± 9.92 years, *p* < 0.01). The mean choroidal thickness of high myopic patients was thinner in the neovascularization group than in the normal group (68.81 ± 48.81 *μ*m vs. 137.80 ± 66.33 *μ*m, *p* < 0.01). The mean choroidal vascular density in the normal group was greater than in the choroidal neovascularization group (82.43 ± 8.73 vs. 67.54 ± 12.56, *p* < 0.01). There was no significant difference in spherical equivalent between the choroidal neovascularization group and the normal group (−10.56 ± 2.97D vs. −11.93 ± 3.38D, *p* = 0.11). Multivariate analysis showed that after adjusting for covariates, less choroidal vascular density and older age were independent factors associated with choroidal neovascularization in the high myopic eye.

**Conclusion:**

Decreased choroidal vascular density and older age played an important role in the development of choroidal neovascularization in high myopic eyes. OCTA may help us to identify the highly myopic patients that need to intervene.

## 1. Introduction

High myopia is defined as a refractive error of more than −6.0 diopters and/or an axial length exceeding 26 mm [1]. As a result of the development of refractive errors and axial length, the retinal layer and choroid layer become thinner. Benavente et al. [2] and Azemin et al. [3] reported that retinal vessel density and choroidal blood flow were decreased in myopia, which could be detected by fundus photography or color Doppler imaging. Alterations in the retina and choroid of high myopia could reduce several pathological structural changes, including lacquer crack formation, chorioretinal atrophy, myopic maculopathy, retinal detachment, and choroidal neovascularization [4–6]. It was reported that the prevalence rate of myopic neovascularization was 10–11% over a period of 12 years [7]. Choroidal neovascularization is a severe lesion that may lead to a dramatic reduction in visual acuity or even blindness.

**Figure 1:**
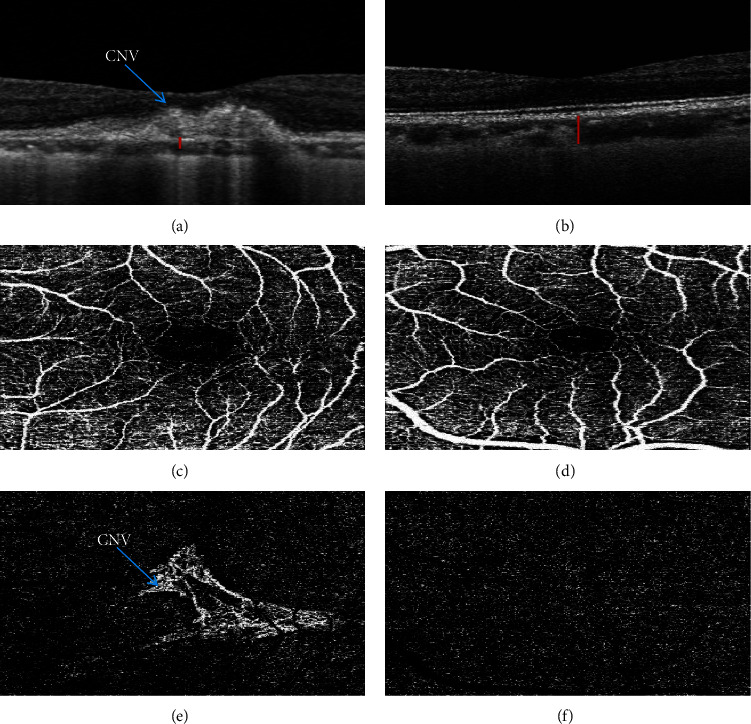
Representative example of swept-source optical coherence tomography (OCTA) images. (a and b) show an OCTA B-scan of high myopia with and without choroidal neovascularization (CNV), respectively. The red lines represent choroidal thickness. (c and d) show the superficial retinal layer on the macula in high myopia with and without CNV, respectively. (e and f) show a deep retinal layer on the macula in high myopia with and without CNV, respectively.

Previous studies of the retinal and choroidal vasculature in high myopic eyes had focused on the use of fundus fluorescence angiography or color Doppler image, which had an invasive nature and experienced poor differentiation of static tissue from the choroidal vasculature [8, 9]. Spectral-domain optical coherence tomography (OCT) is a useful optical technique that provides high resolution and allows visualization of the cross-sectional structure of the retina in vivo [10]. Swept-source optical coherence tomography angiography (OCTA) is an advanced noninvasive imaging technology that can provide 3-dimensional detail of the retinal and choroidal microvasculature [11] (Figure 1). It can be used to quantitatively assess retinal nerve fiber layer thickness, the choroidal thickness, foveal avascular zone, and vessel density. These measurements provide new insights into the pathogenic mechanisms of retinal and choroidal diseases in high myopia eyes. Compared to spectral domain OCT, swept-source OCTA has higher depth penetration and lower signal off that can offer some advantages in the imaging of high myopia with long axial length or deep posterior staphyloma [12].

As we know, several studies have reported OCTA images of choroidal neovascularization arising from age-related macular degeneration and diabetic retinopathy. However, there were very few papers about the choroidal vascular density of choroidal neovascularization in high myopia by using the OCTA. The aim of this study was to analyze choroidal vascular density alteration in high myopia with or without choroidal neovascularization by using OCTA. It could assist us in more clearly understanding the pathophysiological mechanisms of choroidal neovascularization in high myopic eyes.

## 2. Materials and Methods

This was a cross-sectional, observational study about the retinal image performed at the Joint Shantou International Eye Center of the University and the Chinese University of Hong Kong. The Institutional Review Board of the Joint Shantou International Eye Center of Shantou University and the Chinese University of Hong Kong approved the study protocol. All the included subjects provided written informed consent.

### 2.1. Study Subjects

The inclusion criteria were as follows: (1) high myopia, defined as a spherical equivalent refractive error less than 6 diopters and/or axial length equal to or more than 26 mm diopters; (2) age ≥18 years; and (3) high-quality OCTA imaging.

The exclusion criteria included any one of the following: (1) coexisting ocular pathologies such as diabetic retinopathy, glaucoma, age-related macular degeneration, retinal vein occlusion, and retinal vasculitis; (2) having a history of retinal surgery; (3) having type 1 choroidal neovascularization; (4) having poor quality of OCTA images.

**Figure 2:**
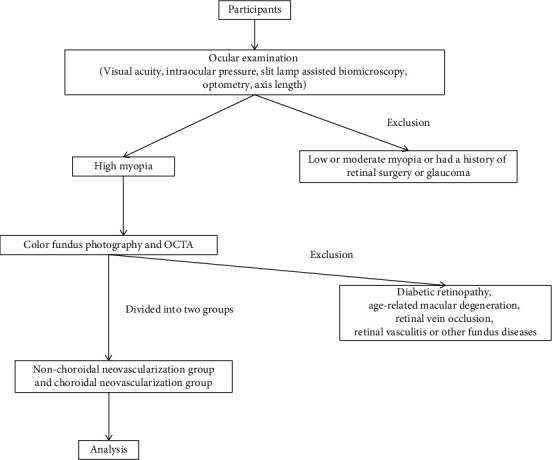
The flow chart of the study population.

All the participants had comprehensive ophthalmic assessments with visual acuity, intraocular pressure, slit lamp-assisted biomicroscopy, color fundus photography, axial length, optometry, and OCTA. Their clinical variables, such as age, sex, and comorbidities, were collected from their medical charts.  Figure 2 shows the flow chart of the study population.

### 2.2. Swept-Source Optical Coherence Tomography Imaging

OCTA scans using the Topcon DRI OCT-1 Atlantis (Capelle aan den Ijssel, The Netherlands). For all the subjects, an experienced, masked investigator examined the retinal and the choroidal segmentations with the angio-retina mode (3 × 3 mm). If the automated positioning or segmentation was deemed inaccurate, manual corrections were performed. Images with substantial motion artifacts were excluded. Choroidal flow density was defined as the percentage area of the choroid occupied by vessels. The choroidal thickness was measured from the retinal pigment epithelium to the sclera on the macular central fovea.

### 2.3. Choroidal Vascular Density Analysis

En-face images of choroidal vasculature were obtained and flattened with the Bruch membrane as a reference by using the en-face tool included in the DRI OCT visualization (Topcon, Tokyo, Japan). The en-face images were exported every 2.6 *μ*m from the Bruch membrane to the choroidal-scleral interface, corresponding to the choroidal thinness, and subsequently imported to ImageJ (National Institutes of Health, Bethesda, Maryland, USA) as an image stack. A mask was created from the image slice at the level of the Bruch membrane, which accounted for the signal from the retinal blood vessels and any minor artifacts. The image stacks and mask were converted to binary images so as to distinguish the choroidal vasculature from the choroidal stoma. To distinguish the choroidal vasculature from the choroidal stoma, we converted the image stacks to binary images by using the ImageJ software. The binarization of the en-face image was carried out using the Otsu method of the software with an automatic threshold-selecting algorithm using gray-level histograms. Then we used the ImageJ command “Process\Noise\Remove Outliers” to remove the noise in the resulting binarized images. The ImageJ command “Process\Image Calculator\Subtract” was performed to eliminate any signal from retinal blood vessels and any minor artifact by applying the mask to all subsequent images. Finally, the choroidal vessels were analyzed by using the ImageJ command “Analyze > Measure.” Also, the percent area occupied by the choroidal vessels for each slice was defined as choroidal vascular density. Then, the average choroidal vascular density of all image slices between the Bruch membrane and the choroidal-scleral interface was recorded [13].

### 2.4. Statistical Analysis

All statistical analyses were performed with SPSS software version 19.0 (SPSS Inc., Chicago, IL). An independent sample *t*-test was used to compare the age, spherical equivalent, choroidal vascular density, and choroidal thickness between normal and patients with choroidal neovascularization. The chi-square test was used to compare the gender and left eye between normal and patients with choroidal neovascularization. Finally, all the variables with a *p*-value <0.05 in univariate analysis were included in logistic regression analysis. The level of significance was defined as a *p*-value less than 0.05.

## 3. Results

60 eyes with high myopia were included in our study, including 30 eyes with choroidal neovascularization and 30 eyes without choroidal neovascularization or other fundus pathology. The characteristics of both groups are summarized in Table 1.

The mean age of high myopic patients was older in the choroidal neovascularization group than in the normal group (48.43 ± 19.06 years vs. 28.83 ± 9.92 years, *p* < 0.01). There was no statistical difference in the left or right eyes between the two groups. There was no statistical significant difference in gender between the two groups.

The mean choroidal thickness of high myopic patients was thinner in the neovascularization group than in the normal group (68.81 ± 48.81 *μ*m vs. 137.80 ± 66.33 *μ*m, *p* < 0.01). The mean choroidal vascular density in the normal group was greater than in the choroidal neovascularization group (82.43 ± 8.73 vs. 67.54 ± 12.56, *p* < 0.01). There was no significant difference in spherical equivalent between the choroidal neovascularization group and the normal group (−10.56 ± 2.97D vs. −11.93 ± 3.38D, *p* = 0.11).

A logistic regression analysis revealed that after adjusting for confounding factors, age (*b* = 0.06 ± 0.03, *p* = 0.02) and choroidal vascular density (*b* = −0.12 ± 0.04, *p* = 0.01) were still significantly associated with choroidal neovascularization (Table 2).

## 4. Discussion

Our study found that high myopic patients with older age, thinner choroidal thickness, and less choroidal vascular density were strongly associated with choroidal neovascularization in univariate analysis. Multivariate analysis showed that after adjusting for covariates, less choroidal vascular density and older age were independent factors associated with choroidal neovascularization in high myopic patients.

Our study discovered that choroidal thicknesses were thinner in high myopic eyes with choroidal neovascularization. This result was consistent with previous studies. It was reported that choroidal thickness changed in response to imposed myopic or hyperopic defocusing in a bidirectional manner [14–17]. Zhang et al. [18] discovered that choroidal thickness was significantly decreased in guinea pig myopia. Vincent et al. [19] examined the choroidal thickness in a sample of myopic anisometropia and found the choroidal thickness was significantly thinner in the more myopic eyes. Liu et al. [20] found that choroidal thickness was thinner in anisometropic myopic eyes. The choroid is a highly vascularized tissue consisting of the choriocapillaris and medium- and large-vessel layers. There have been studies reporting that losses in Haller's and Sattler's layers lead to choroidal thinning in myopic eyes [21, 22]. The choriocapillaris is supplied by Haller's and Sattler's layers, so reducing blood flow in these layers can damage choriocapillaris blood perfusion. So, choroid thickness reduction may be accompanied by decreased choroidal blood flow. This was consistent with our result that decreased choroidal vascular density was associated with choroidal neovascularization in high myopia. However, Nishida et al. [23] found that there was no significant functional compromise in some myopic eyes with extremely thin choroid thickness. It is possible that the choriocapillaris perfusion area may be maintained by choroidal microcircular autoregulation in the early myopia stage [24].

As we know, this is the first report about the alteration of choroidal vascular density due to choroidal neovascularization in high myopia by using OCTA. We found that lower choroidal vascular density plays an important role in the development of choroidal neovascularization in high myopic eyes. There were a few studies that disagreed with our results. Mo et al. [25] found that there was no significant difference in choriocapillaris flow density between high myopia and pathological myopia. This difference in the literature could be attributable to the relatively narrow age ranges of patients. However, there were more studies that supported our results. Liu et al. [26] reported the choroidal vascularity index was significantly lower and the with choroidal layer thinner in high myopia eyes than in low to moderate myopia eyes. They concluded that the choroidal vascularity index may help to discover the underlying pathophysiology of nonpathological highs. However, their participants were all nonpathological myopic patients, which was different from this study. A study comprising 44 participants with anisometropic myopia found the vascular density of the choriocapillaris was reduced in the more myopic eyes [20]. Su et al. [27] studied the choriocapillaris in myopic eyes by using OCTA and found the choriocapillaris had greater impairment in eyes with high myopia, especially in the absence of features of pathologic or degenerative myopia. It is reported that poor blood circulation may result in neovascularization [28–30]. Liu et al. [31] found that choroidal neovascularization in high myopia was surrounded by an area with insufficient blood vessels on the choroid capillary layer. There was evidence showing that vascular endothelial growth factor release and consequent myopic choroidal neovascularization attributed to decreased choroidal blood flow over a prolonged period of time [32]. It was reported that actively increasing choroidal blood flow with the vasodilator prazosin inhibited axial elongation and scleral hypoxia in guinea pig myopia [33]. Looking forward to the future, safe treatments for humans should be developed to increase the choroidal blood flow in high myopic eyes and prevent the development of choroidal neovascularization.

Our study also found older high-myopia participants were more likely to develop choroidal neovascularization. This result was consistent with much previous literature. A 10-year follow-up study of high myopic patients with or without maculopathy showed the prognosis of patients with myopic maculopathy worsened associated with older age [34]. Yu et al. [35] reported a significant negative correlation between the blood perfusion in the macular and age by using OCTA to analyze participants over 35 years old. Burgansky-Elias et al. [36] also reported that older age was associated with a greater reduction of flow velocity in the venule of participants over the age of 40 years. Zheng et al. [37] confirmed that choroidal vessels perfusion deficits in the macula worsened with older age.

Our study showed no significant difference in spherical equivalent between the choroidal neovascularization group and the normal group. This interesting finding was confirmed by previous studies. Jiang et al. [9] found that there was no difference in vascular densities of the choriocapillaris between the high myopic eye and the nonhigh myopic eye. A cross-sectional study including 154 eyes from 77 young myopic adults found the values of the choriocapillaris perfusion area did not correlate significantly with spherical equivalent or axial length [24]. Flores-Moreno et al. [38] also reported that the density of the choriocapillaris was not associated with axial length, although the choroidal thickness was dependent on axial length.

Limitations to our study included elongation of axial length in high myopic eyes, which led to a different magnification of OCTA images and impacted the accuracy of layer division by the OCTA system automatically. The OCTA systems cannot provide a method of axial length correction [39], so the application of magnification correction was needed to minimize errors resulting from image distortion.

## 5. Conclusion

Overall, this study found that choroidal thicknesses were thinner in high myopic eyes with choroidal neovascularization than in normal high myopic eyes. This paper also discovered that lower choroidal vascular density and older age play an important role in the development of choroidal neovascularization in high myopic eyes. Another interesting result of this study was that there was no significant difference in spherical equivalent between the choroidal neovascularization group and the normal group. Consequently, new treatments should be developed to increase the choroidal blood flow in high myopic eyes and prevent the development of choroidal neovascularization. Also, OCTA may help us to identify the highly myopic patients that need to intervene.

## Figures and Tables

**Table 1:** Comparison of clinical characteristics between the included high myopia eyes with or without choroidal neovascularization.

Factors	Nonchoroidal neovascularization	Choroidal neovascularization	*p*-value
Number	30	30	
Gender			0.793^*∗*^
Male	12 (40%)	13 (43%)	
Female	18 (60%)	17 (57%)	
Age (years)	28.83 ± 9.92	48.43 ± 19.06	<0.01^#^
Spherical equivalent (*D*)	−10.56 ± 2.97	−11.93 ± 3.38	0.11^#^
Left eye	12 (40%)	19 (63%)	0.07^*∗*^
Choroidal vascular density	82.43 ± 8.73	67.54 ± 12.56	<0.01^#^
Choroidal thickness (*μ*m)	137.80 ± 66.33	68.81 ± 48.81	<0.01^#^

^#^: independent sample *t*-test; ^*∗*^: chi-square test.

**Table 2:** Multivariate analysis for clinical characteristics for the included high myopia eyes with or without choroidal neovascularization.

Factors	*B* ± S.E.	*p*-value
Age (years)	0.06 ± 0.03	0.02
Choroidal vascular density	−0.12 ± 0.04	0.01
Choroidal thickness (*μ*m)	—	0.30

## Data Availability

The data used to support the findings of this study are available from the corresponding author upon request.
